# Assessing the role of low-emission hydrogen: A techno-economic database for hydrogen pathways modelling

**DOI:** 10.1016/j.dib.2023.109822

**Published:** 2023-11-20

**Authors:** F.A. Plazas-Niño, R. Yeganyan, C. Cannone, M. Howells, B. Borba, J. Quirós-Tortós

**Affiliations:** a Industrial and Business Studies School, Universidad Industrial de Santander, Bucaramanga, Colombia; b Centre for Environmental Policy, Imperial College London, London, United Kingdom; c STEER Centre, Department of Geography, Loughborough University, Loughborough, United Kingdom; d Electrical Engineering Department, Federal Fluminense University (UFF), Niteroi, RJ, Brazil; e School of Electrical Engineering, University of Costa Rica, San José, Costa Rica

**Keywords:** Energy system modelling, Energy transition, Hydrogen economy, OSeMOSYS, Energy policy

## Abstract

Hydrogen is globally acknowledged as a versatile energy carrier crucial for decarbonization in multiple sectors. Many countries have initiated the development of national hydrogen roadmaps and strategies, recognizing hydrogen as a strategic resource for achieving sustainable energy transitions. Formulating these guidelines for future action demands a solid technical foundation to facilitate well-informed decision-making. Energy system modelling has emerged as a significant scientific tool to assist governments and ministries in designing hydrogen pathways assessments based on scientific outcomes. The first step in the modelling process involves gathering, curating, and managing techno-economic data, a process that is often time-consuming and hindered by the unavailability and inaccessibility of data sources. This paper introduces an open techno-economic dataset encompassing key technologies within the hydrogen supply chain, spanning from production to end-use applications. Energy modelers, researchers, policymakers, and stakeholders can leverage this dataset for energy planning models, with a specific focus on hydrogen pathways. The presented data is designed to promote modelling studies that are retrievable, reusable, repeatable, reconstructable, interoperable, and auditable (U4RIA^1^). This enhanced transparency aims to foster greater public trust, scientific reproducibility, and increased collaboration amongst academia, industry, and government in producing technical reports that underpin national hydrogen roadmaps and strategies.

Specifications Table

**Table:** 

Subject	Energy
Specific subject area	Hydrogen Pathways Modelling
Data format	Raw Processed
Type of data	Table
Data collection	Literature survey (databases, reports from international organizations, reports from national institutions, peer-reviewed journal articles)
Data source location	Raw data sources are listed in the different sections of this article
Data accessibility	With this article and in a repository Repository name: Mendeley Data - Techno-Economic Energy Dataset for Hydrogen Pathways Modelling Data identification number: 10.17632/8ztnn4br66.1 Direct URL to data: https://data.mendeley.com/datasets/8ztnn4br66/1

## 1 Value of the Data

• The dataset covers the entire hydrogen system, spanning from production to demand, which is not common in current literature.
• This dataset can be used to model hydrogen pathways using a techno-economic model. Although originally designed for implementation in an OSeMOSYS model, the data is valuable for any energy system model.
• Researchers, practitioners, and the scientific community can employ this dataset for analysis in the development of hydrogen roadmaps, assessments, strategies, or any other technical documents.
• The structure of this dataset can set a standard for similar studies in hydrogen pathways modelling, promoting the use of open data principles.

## 2 Background

This open dataset is expected to serve as an accessible resource for the entire scientific community interested in hydrogen pathways for energy system modelling. With its combination of transparency and technical rigour, our dataset has the potential to enhance the science-policy interface and, consequently, policymaking effectiveness. This work aligns with the U4RIA goals [1], which encompass Ubuntu, Retrievability, Reusability, Repeatability, Reconstructability, Interoperability, and Auditability. Furthermore, this dataset aims at setting a first iteration of an open dataset focused on hydrogen technologies.

## 3 Data Description

**Fig. 1:**
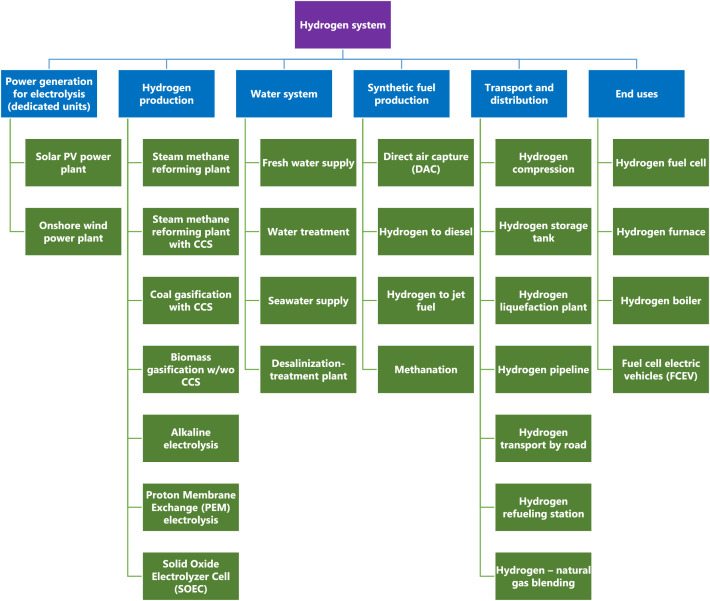
Structure of the dataset covering 6 categories from hydrogen production to end uses of hydrogen.

The data described in this paper is a new addition to a first iteration of an open dataset for the assessment of decarbonization pathways [2]. The focus is to provide a more detailed dataset to model hydrogen pathways. The structure of the dataset is designed to align with the open source energy modelling system (OSeMOSYS) [3], however, the techno-economic data is applicable to any modelling framework. The data presented was gathered from reports, websites, and datasets of international and national organizations, and from peer-reviewed journal papers. The data encompasses capital costs, fixed costs, variable costs, operational lifetimes, efficiencies and capacity factors. The dataset is openly accessible in a Mendeley Data Repository following the next link: https://data.mendeley.com/datasets/8ztnn4br66/1. The dataset covers 36 technologies spanning from hydrogen production to end uses of hydrogen in transport and industry. Fig. 1 summarizes the structure of the dataset with the list of principal technologies per category.

### 3.1 Costs

Costs are categorized into three groups: capital costs, fixed costs, and variable costs. The timeseries data covers the period from 2021 to 2050. The data projections are based on a literature review when available; otherwise, constant values are assumed in the absence of information. An excerpt of the costs for a sample of four technologies in key years is presented in Table 1. The full dataset is accessible in the Excel file ‘DATASET H2′ in the repository.

**Table 1:** Costs of technologies for key years (excerpt).

Cost	Unit	Technology	2021	2030	2040	2050
Capital cost	MUSD/(PJ/year)	Steam Methane Reforming plant + CCS	34.08	32.9	31.64	30.43
		Coal gassification+CCS	92.56	92.56	92.56	92.56
		Biomass gassification+CCS	102.93	102.93	102.93	102.93
		PEM electrolyser	36.75	27.55	20	14.52
Fixed cost	MUSD /(PJ/year)	Steam Methane Reforming plant + CCS	1.36	1.31	1.26	1.21
		Coal gassification+CCS	4.63	4.63	4.63	4.63
		Biomass gassification+CCS	9.67	9.67	9.67	9.67
		PEM electrolyser	1.16	1.12	1.06	1.02
Variable cost (non-fuel)	MUSD/PJ	Steam Methane Reforming plant + CCS	2.73	2.73	2.73	2.73
		Coal gassification+CCS	5.96	5.96	5.96	5.96
		Biomass gassification+CCS	7.65	7.65	7.65	7.65
		PEM electrolyser	0	0	0	0

### 3.2 Technical parameters

The dataset covers three crucial technical parameters: operational lifetime, efficiency, and capacity factor. These parameters play a fundamental role in quantifying technology capacity expansion within energy system modelling. Operational lifetime represents the number of years a technology remains useful. Efficiency measures the relationship between delivered energy and input energy. Capacity factor is a key sizing factor that expresses the ratio between delivered energy and the potential energy production under full operational conditions. You can find comprehensive definitions for each parameter in [2,4]. Table 2 provides an excerpt of the technical parameters for a sample of technologies. In cases where data was unavailable, efficiency and capacity factor were assumed to be equal to 1. The complete dataset is accessible in the Excel file ‘DATASET H2’ in the repository.

**Table 2:** Technical parameters for a sample of technologies (excerpt).

Technology	Operational lifetime	Efficiency	Capacity factor
Onshore wind power plant	30	1	0.34
Alkaline electrolyser	30	0.769	0.98
Desalinization-treatment plant	30	12.96 PJ electricity/BCM	1
Direct air capture (DAC)	20	10.26 PJ electricity/MtCO2	0.95
Hydrogen compression	15	0.995	1
Hydrogen refuelling station	30	1	1
Hydrogen fuel cell	30	0.6	1
Hydrogen light duty vehicle (FCEV)	17	0.827 PJ/Gpkm	1

## 4 Experimental Design, Materials, and Methods

The dataset was thoroughly curated through an extensive literature review, which included reports, websites, datasets from international and national organizations, and peer-reviewed journal papers. The raw data was pre-processed to standardize units, ensuring dimensional uniformity for the modelling workflow. Details regarding the data sources and assumptions used in constructing the dataset are provided in the following sections.

### 4.1 Costs

Cost data were sourced from various references, as outlined in Table 3. Cost projections were extracted from the literature and were not calculated. When projected data was not available, constant values were assumed. All costs were standardized to 2021 dollars using the average euro-dollar exchange rate from [5] and the inflation rate based on [6]. For carbon capture and storage (CCS) technologies, we incorporated a variable cost equal to 36.1 US$/t [7] to account for the financial cost of infrastructure for transport and storage of CO_2_.

**Table 3:** List of sources for cost data per category.

Category	References
Power generation for electrolysis	[8]
Hydrogen production	[9], [10], [11], [12]
Water system	[13,14]
Synthetic fuel production	[15,16]
Transport and distribution	[9,^15^,17]
End uses	[18], [19], [20]

### 4.2 Technical parameters

Table 4 provides a summary of the literature sources used to compile the technical data for this study. When data for operational lifetimes was not available, values of analogous technologies were assumed. For fuel cell electric vehicles (FCEV), we estimated lifetimes using the average ages of vehicles in Colombia as a reference point for a developing country [21]. In the case of virtual technologies such as 'freshwater supply' and 'natural gas-hydrogen blending,' a default lifetime of 100 years is assumed. For transport and distribution technologies, we assumed an efficiency of 1 due to a lack of data. Similarly, capacity factors are set to 1 for all technologies when no data was available.

**Table 4:** List of sources for technical data per category.

Category	References
Power generation for electrolysis	[8,22]
Hydrogen production	[9], [10], [11], [12],23]
Water system	[13,14]
Synthetic fuel production	[15,16]
Transport and distribution	[9,^15^,^17^,24]
End uses	[18], [19], [20]

In the case of blending, we assumed a 10 % volumetric blending of hydrogen with natural gas. To facilitate its use in energy system modelling, we applied Eqs. (1) and 2. These equations allowed us to convert the blending ratio from volume to energy units. By considering the energy density of natural gas (e.g., 0.001009557 PJ/Mcf) and hydrogen (e.g., 0.000305822 PJ/Mcf), we determined the corresponding blending ratios in energy units. It's crucial to ensure that the units of energy density match to maintain the non-dimensionality of the ratios.

(1) $$Ratio\;Hydrogen-blending=\frac{P*EH}{P*EH+\left(1-P\right)*EG}$$

(2) $$Ratio\;Natural\;gas-blending=\frac{\left(1-P\right)*EG}{P*EH+\left(1-P\right)*EG}$$

Where

*P = Percentage of blending indecimal formEH = Energy density of hydrogen* $\left(\frac{energy}{volume}\right)$ *EG = Energy density of natural gas* $\left(\frac{energy}{volume}\right)$

## Limitations

Not applicable.

## Ethics Statement

Not applicable.

## CRediT authorship contribution statement

**F.A. Plazas-Niño:** Conceptualization, Methodology, Formal analysis, Investigation, Data curation, Writing – original draft. **R. Yeganyan:** Project administration, Writing – review & editing. **C. Cannone:** Writing – review & editing. **M. Howells:** Supervision. **B. Borba:** Writing – review & editing. **J. Quirós-Tortós:** Supervision, Writing – review & editing.

## Data Availability

Techno-Economic Energy Dataset for Hydrogen Pathways Modelling (Reference data) (Mendeley Data)
